# Whole Genome Classification and Phylogenetic Analyses of Rotavirus B strains from the United States

**DOI:** 10.3390/pathogens7020044

**Published:** 2018-04-18

**Authors:** Frances K. Shepherd, Diana Maria Herrera-Ibata, Elizabeth Porter, Nitipong Homwong, Richard Hesse, Jianfa Bai, Douglas G. Marthaler

**Affiliations:** 1 Department of Veterinary Biomedical Sciences, College of Veterinary Medicine, University of Minnesota, St. Paul, MN 55108, USA; sheph085@umn.edu; 2 Veterinary Diagnostic Laboratory, College of Veterinary Medicine, Kansas State University, Manhattan, KS 66506, USA; dianaherrera@vet.k-state.edu (D.M.H.-I.); epoulsen@vet.k-state.edu (E.P.); dhesse@vet.k-state.edu (R.H.); jbai@vet.k-state.edu (J.B.); 3 Diagnostic Medicine/Pathobiology, College of Veterinary Medicine, Kansas State University, Manhattan, KS 66506, USA; 4 Department of Animal Science, Kasetsart University, Kamphaeng Saen Campus, Kamphaeng Saen, Chatuchak, Bankok 10900, Thailand; nitipong.h@ku.ac.th

**Keywords:** rotavirus B virus, phylogenetic analysis, classification, whole genome sequencing

## Abstract

Rotaviruses (RVs) are a major etiological agent of acute viral gastroenteritis in humans and young animals, with rotavirus B (RVB) often detected in suckling and weaned pigs. Group A rotavirus classification is currently based on the two outer capsid proteins, VP7 and VP4, and the middle layer protein, VP6. Using RVB strains generated in this study and reference sequences from GenBank, pairwise identity frequency graphs and phylogenetic trees were constructed for the eleven gene segments of RVB to estimate the nucleotide identity cutoff values for different genotypes and determine the genotype diversity per gene segment. Phylogenetic analysis of VP7, VP4, VP6, VP1–VP3, and NSP1–NSP5 identified 26G, 5P, 13I, 5R, 5C, 5M, 8A, 10N, 6T, 4E, and 7H genotypes, respectively. The analysis supports the previously proposed cutoff values for the VP7, VP6, NSP1, and NSP3 gene segments (80%, 81%, 76% and 78%, respectively) and suggests new cutoff values for the VP4, VP1, VP2, VP3, NSP2, NSP4, and NSP5 (80%, 78%, 79%, 77% 83%, 76%, and 79%, respectively). Reassortment events were detected between the porcine RVB strains from our study. This research describes the genome constellations for the complete genome of Group B rotaviruses in different host species.

## 1. Introduction

Rotaviruses (RVs) are a major etiological agent causing acute viral gastroenteritis in humans and young animals, including young calves, weaning and post-weaning pigs [1,2]. RVs are members of the *Reoviridae* family, with a genome consisting of eleven double-stranded RNA gene segments that encode six structural proteins (VP1–VP4, VP6, and VP7) and five or six nonstructural proteins (NSP1–NSP5/NSP6) [3]. The triple-layered capsid is comprised of the outer layer of VP7 and VP4, the inner layer of VP6, and the core VP2. RVs are classified into eight species (*A-H*) based on antigenic relatedness or sequencing of the inner capsid protein VP6. Two tentative species, I and J, have recently been identified in fecal specimens from sheltered dogs in Hungary and guano samples from bats from Serbia, respectively [4,5,6]. The most common species infecting animals, including humans, are rotavirus A, B and C (RVA, RVB, and RVC, respectively), with RVA being the most prevalent, whereas Groups D–H only infect animals [3,7].

Rotavirus B (RVB) was first identified as the cause of severe gastroenteritis among adults in China from late 1982 to early 1983. RVB continued to be responsible for diarrheal disease in humans in India, Bangladesh, Nepal, and Myanmar [8,9,10,11,12,13,14,15]. In addition to humans, RVB strains have been detected in different host species such as rats [16], cattle [17,18,19,20,21,22], goats [23,24], sheep [25], and swine [26,27,28]. RVB has yet to be isolated in cell culture, which has hampered obtaining serological information on this species.

RVA strains have been well characterized, and, currently, the Rotavirus Classification Working Group (RCWG) maintains an RVA classification system calculating percent identity cutoff values to categorize the genotypes for the eleven segments of RVA [29]. Genotype constellations are denoted as Gx-P[x]-Ix-Rx-Cx-Mx-Ax-Nx-Tx-Ex-Hx representing the VP7-VP4-VP6-VP1-VP2-VP3-NSP1-NSP2-NSP3-NSP4-NSP5/6 encoding genes, respectively. The RVB percent identity cutoff values of 80%, 81%, 70%, 76%, 75%, 78%, 70% and 78% have been established for segments VP7, VP6, VP3, NSP1, NSP2, NSP3, NSP4, and NSP5, respectively [28,30,31,32,33,34]. Based on these cutoff values, 24 G [35], 13 I [31], 4 M [30], 7 A [34], 4 N [32], 4 T [30], 4 E [30], and 6 H [33] genotypes have been identified. In the present study, RVB nucleotide sequences of several porcine strains and a single caprine strain were determined to investigate the genetic diversity and illustrate the phylogenetic relationships of all 11 RVB gene segments to identify distinct genotypes and reassortment events.

## 2. Materials and Methods

Extraction of RNA from clinical samples used Trizol reagent (Ambion, Carlsbad, CA, USA) and DirectZol filter columns (Zymo Research, Irvine, CA, USA). Full-length cDNA was produced by the single primer amplification technique (SPAT) from dsRNA [36,37]. Briefly, DNA primers were ligated onto the 3′ ends of the double-stranded RNA genome segments, and RT-PCR was carried out using primers complementary to the ligated sequences. The cDNA was prepared for next-generation sequencing (NGS) with the NexTera XT library preparation kit (Illumina, San Diego, CA, USA). Sequencing was performed on the Miseq (Illumina) NGS platform using the 2 × 150 bp run option. Raw de-multiplexed sequencing reads were trimmed and de novo assembled using the CLC Genomics Workbench (Qiagen Bioinformatics/CLC Bio, Redwood City, CA, USA). The complete CDS for the NSP genes were obtained, whereas some of the VP genes for specific strains could not be obtained (VP4 and VP2 from strain RVB/Pig-wt/USA/KS2/2012; VP1 and VP3 from strain RVB/Goat-wt/USA/CA22/2014) due to a low viral read count. The porcine and caprine RVB nucleotide sequences used in the present study have been submitted to GenBank (NCBI) and under the following accession numbers: NSP1 (MF966596–MF966617), NSP2 (MG271985–MG272006), NSP3 (MG272007–MG272028), NSP4 (MG272029–MG272050), NSP5 (MG272051–MG272072), VP1 (MG272073–MG272093), VP2 (MG272094–MG272114), VP3 (MG272115–MG272135), VP4 (MG272136–MG272161), VP6 (MG272162–MG272183), and VP7 (MG272184–MG272206).

The RVB sequences from this study and the RVB sequences available from the GenBank were aligned using Muscle alignment in Geneious 10.1.3 software [38]. Strains with less than 80% of the open reading frame were excluded from analysis. To determine the genotype classification of the eleven dsRNA segments, phylogenetic trees and pairwise nucleotide (nt) identity frequency graphs were created, and the cutoff values were defined as the percentages separating nucleotide identities between inter and intra genotypes [29]. Kruskal–Wallis chi-squared rank sum test was run to determine host nucleotide identities difference per gene segment. The phylogenetic trees were constructed by maximum likelihood using general time reversible substitution model [39] with 500 bootstrap replicates in Geneious software. 

## 3. Results

Porcine fecal samples (*n* = 21) from farms in Illinois and Kansas and a single goat fecal sample from California were submitted to the Veterinary Diagnostic Laboratory at Kansas State University between 2012 and 2014 for sequencing. Pairwise identity frequency graphs (Supplementary Materials, Figure S1) and phylogenetic trees were constructed for the eleven gene segments of RVB strains generated in the present study and RVB sequences available from GenBank to assess nucleotide identity between the RVB host species and determine the nucleotide percent identity cutoff values and number of genotypes per gene segment. Porcine median nucleotide identities were significantly lower than bovine and human for all gene segments except VP1, VP2, NSP1, NSP2, and NSP3 (Table 1). Human strains had the highest median nucleotide identities for all but the VP3, NSP1, NSP3, NSP4, and NSP5 segments.

**Figure 1:**
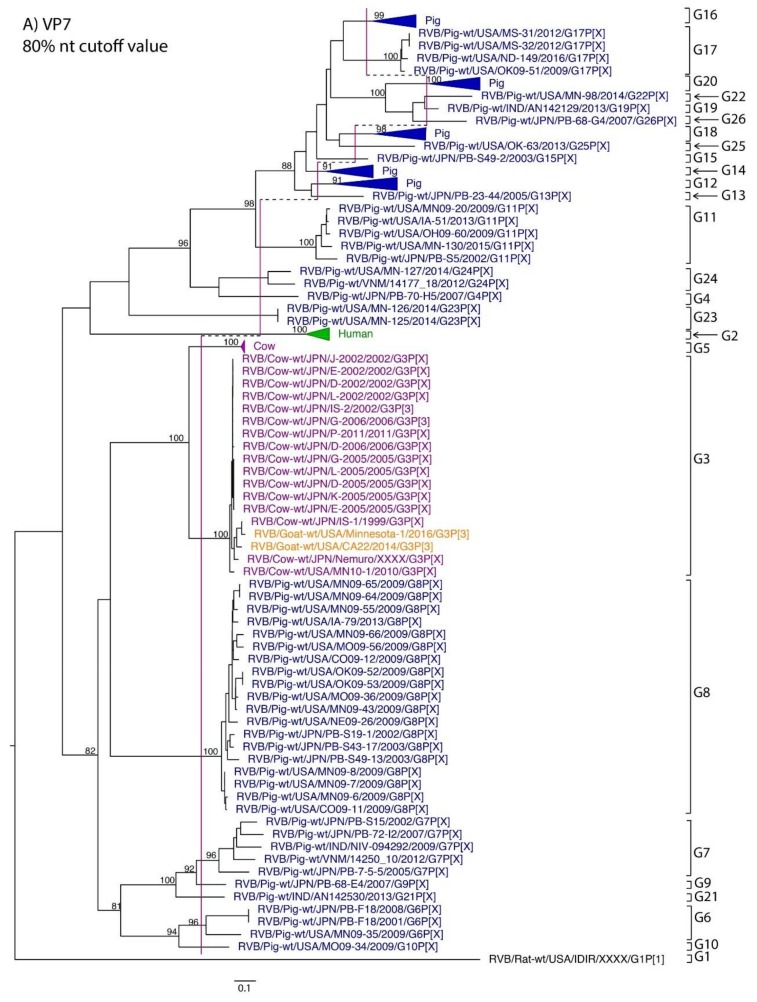

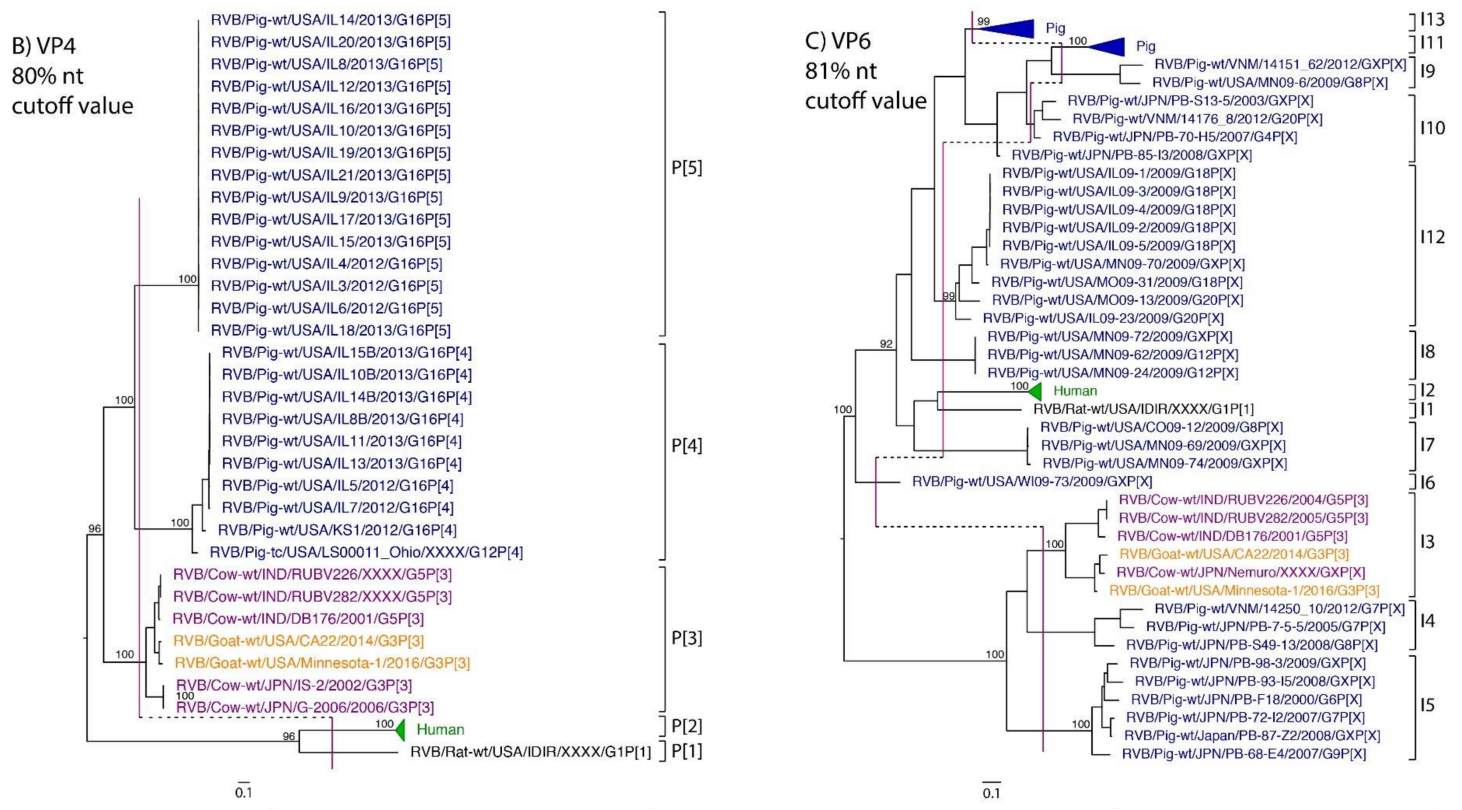

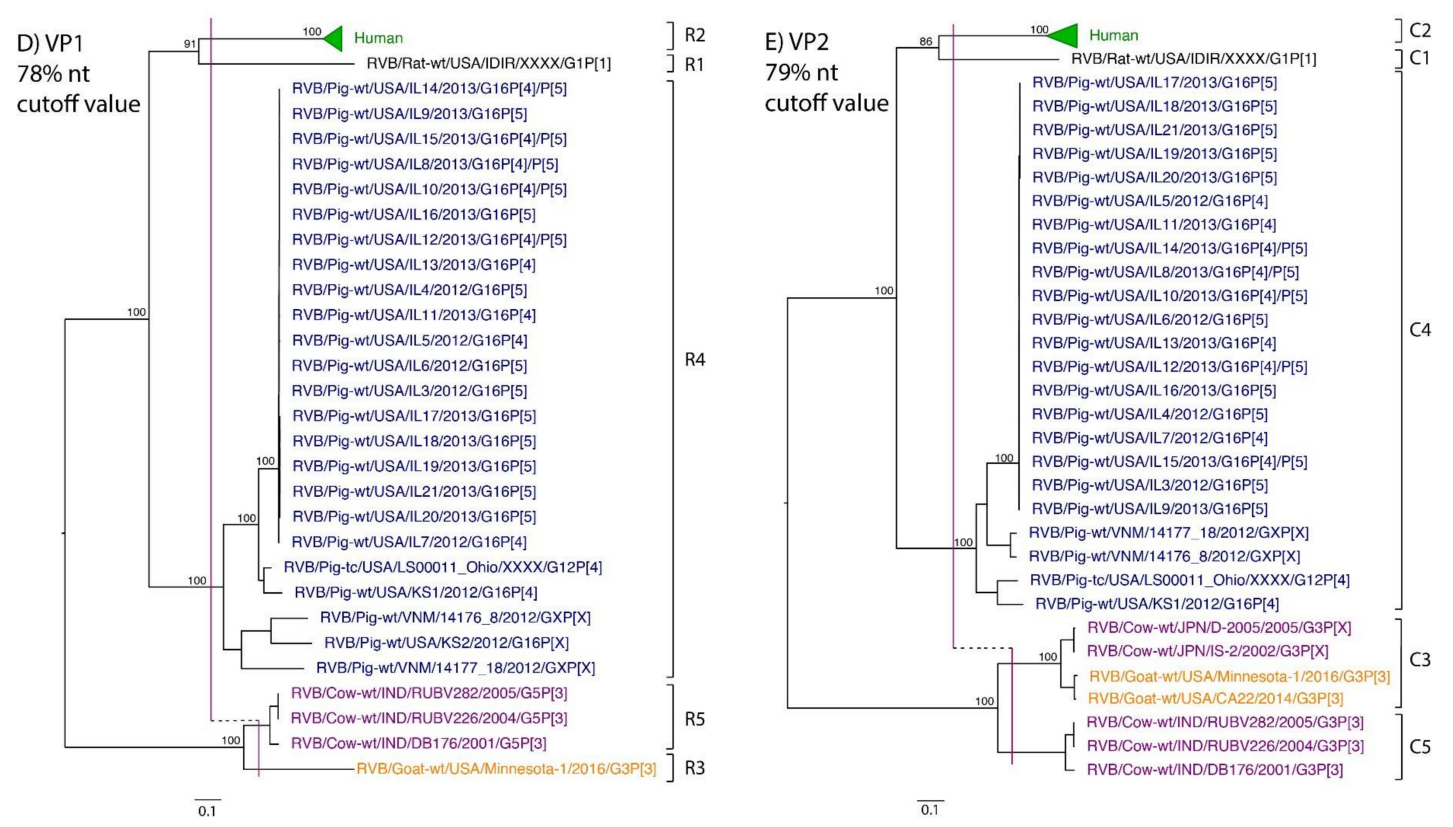

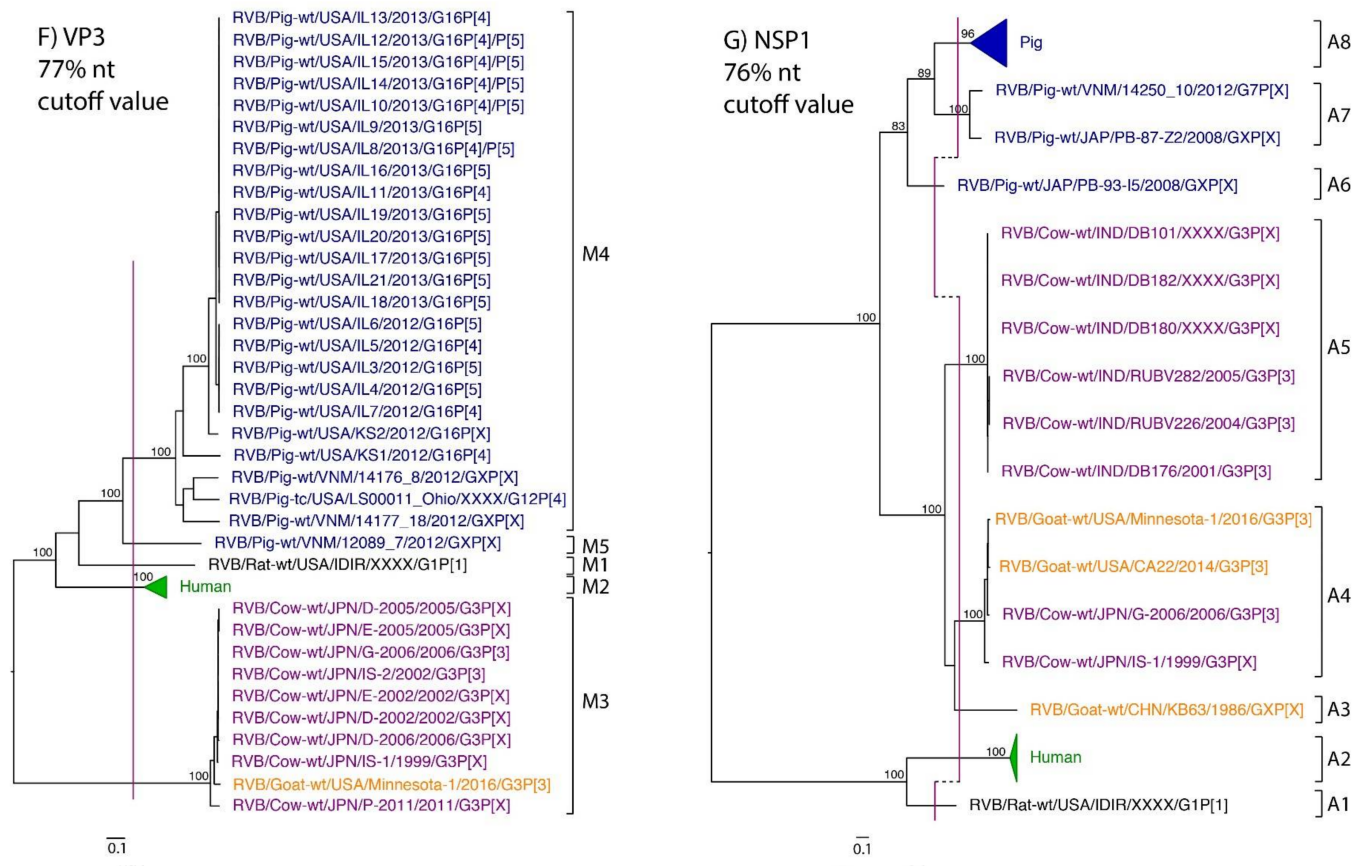

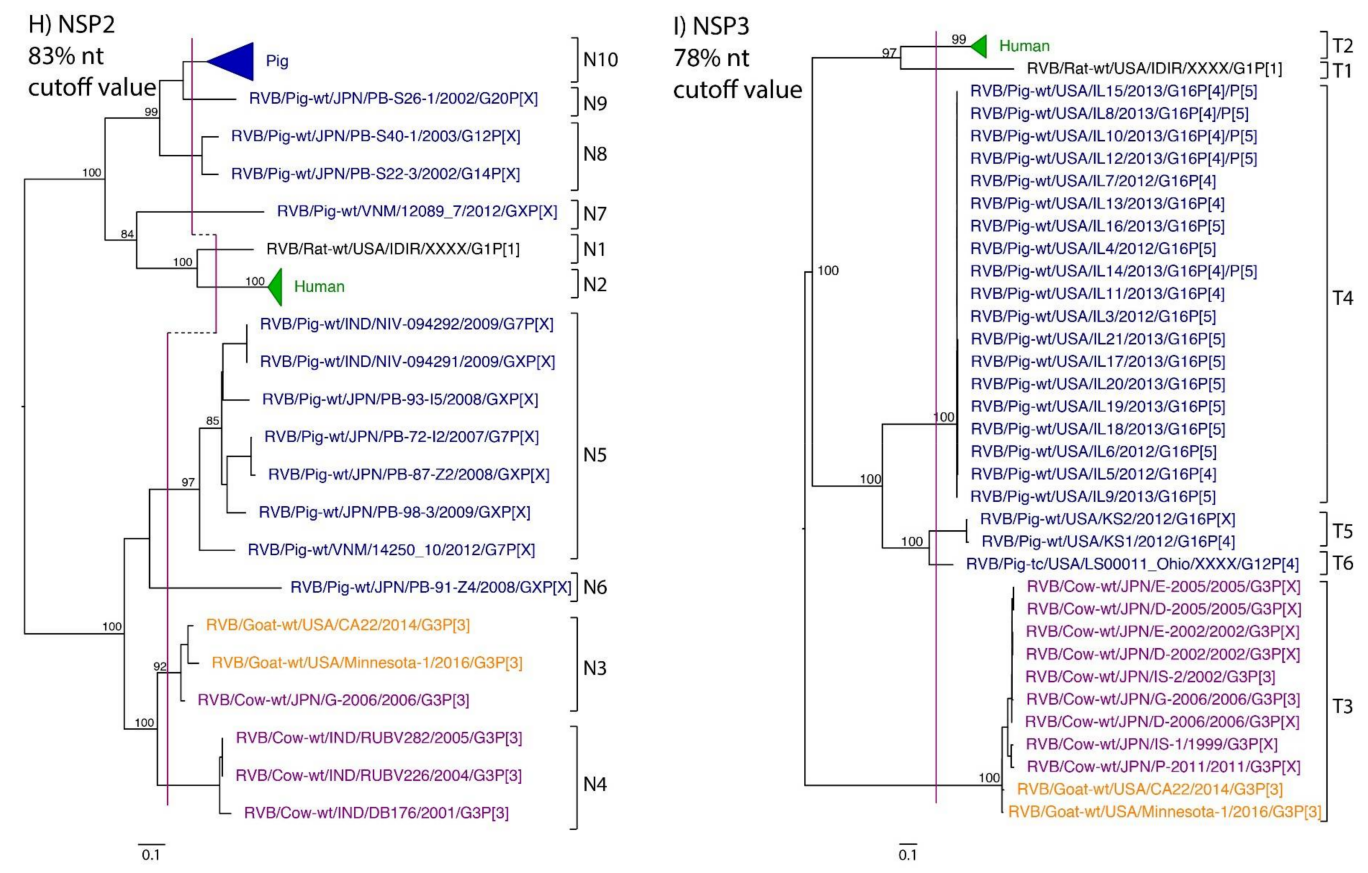

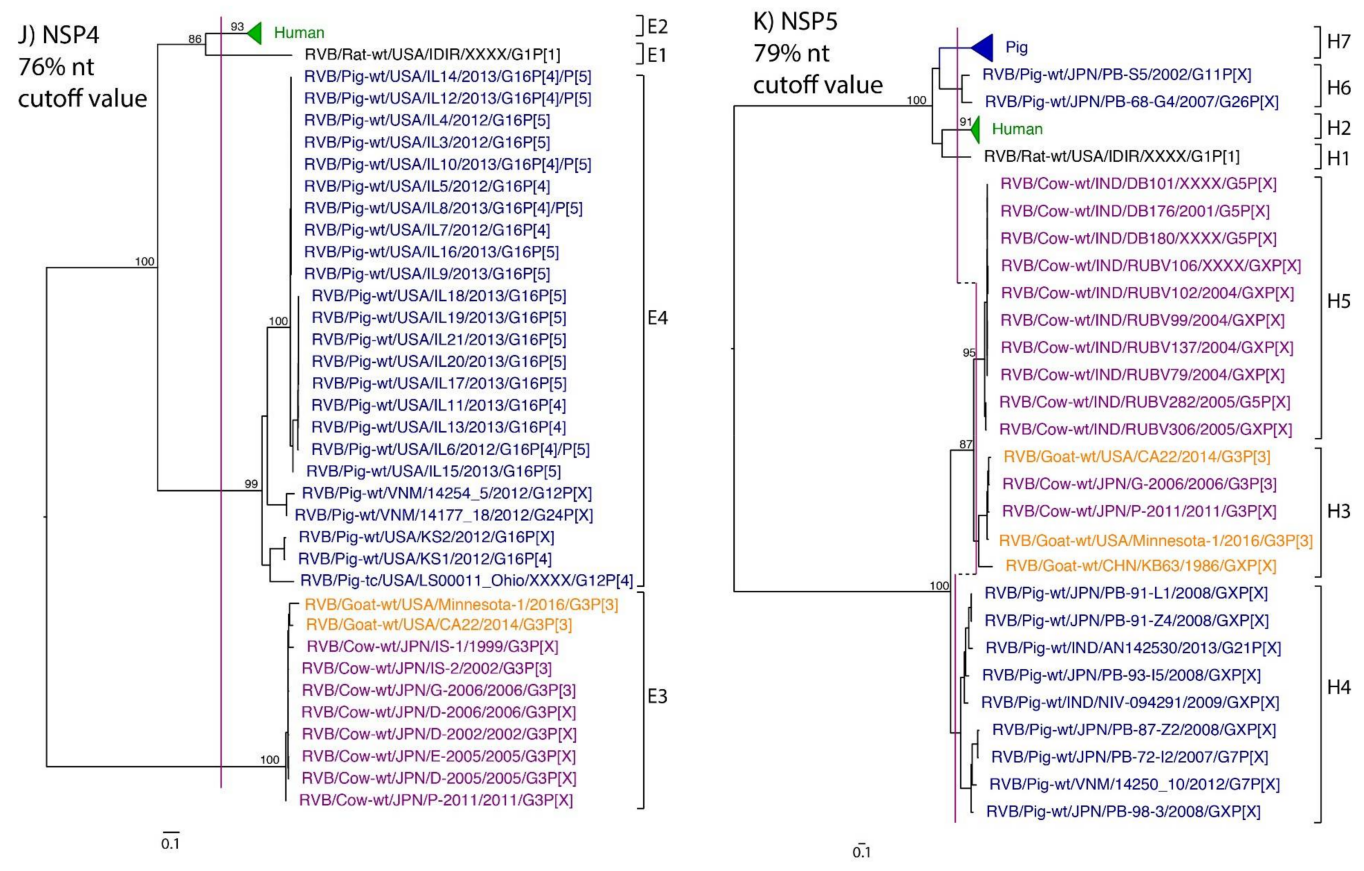
Phylogenetic trees of the 11 gene segments of RVB (**A**–**K**). Bootstrap percentages (per 500 replicates) are shown at nodes. Values below 80% are not shown. Sequences are colored according to host species. Dashed lines represent the nucleotide (nt) cutoff value with genotypes labeled by brackets.

While nt cutoff values identified in this study were consistent with already established cutoffs for the VP7, VP6, NSP1, and NSP3 gene segments (80%, 81%, 76% and 78%, respectively), pairwise identity frequency graphs indicated new nt cutoff values for the NSP2, NSP4, NSP5 and VP3 (83%, 76%, 79% and 77%, respectively; Table 2). In addition, we propose nt cutoff values of 80%, 78% and 79% for VP4, VP1, and VP2, respectively. The NSP5 gene segment includes an additional genotype to the six genotypes reported by [33], classifying the caprine strains and the Japanese bovine strains within the same genotype (Figure 1). Phylogenetic analysis showed no interspecies mixing of genotypes among porcine, human, or murine strains while goat and bovine strains shared clades for all segments except VP1.

Murine, human and caprine RVB strains had conserved genome constellations of G1-P[1]-I1-R1-C1-M1-A1-N1-T1-E1-H1, G2-P[2]-I2-R2-C2-M2-A2-N2-T2-E2-H2, and G3-P[3]-I3-R3-C3-M3-A3-N3-T3-E3-H3, respectively (Table 3). The Japanese bovine strains and the US caprine strains belong to the same genotypes for the available gene segments. The porcine strains from Kansas show a genome constellation of G14/G16-P[4]-I13-R4-C4-M4-A8-N10-T5-E4-H7. The Illinois strains share the same genome constellation of G16-P[5]-I13-R4-C4-M4-A8-N10-T4-E4-H7 except for the gene segment VP4, which contains P[4] and P[5] genotypes. Reassortment among gene segments of porcine strains was indicated by the conserved genotypes G16, I13, R4, C4, M4, A8, N10, E4, and H7 associating with multiple VP4 and NSP3 (P[4]/P[5] and T4/T5, respectively). 

## 4. Discussion

Until now, genotype classifications for the RVB gene segments VP1, VP2, and VP4 were lacking. This study identified percent identity nucleotide cutoff values for VP1, VP2, and VP4 while updating VP3, NSP2, NSP4 and NSP5 cutoff values using additional porcine RVB strains from the US. Compared to RVA and RVC, the RVB nucleotide percent identity cutoff values are lower for all gene segments except for the VP7, which shares the same nucleotide cutoff value with RVA [29,40]. The lower cutoff values suggest higher sequence diversity of RVB compared to other rotavirus species, which has been discussed in previous studies as well [22,27]. Refuted by more recent studies illustrating that the range of percent identities of VP6 are between 65% and 100% for both RVA and RVB [4], RVB does appear to be more diverse when considering the number of genotypes present in certain hosts. In swine, only three and eight VP6 genotypes have been identified for RVA and RVC, respectively, compared to ten VP6 genotypes for RVB [31,40,41]. There are 17 VP7 genotypes of RVB in swine compared to 12 and 15 VP7 genotypes in RVA and RVC, respectively [42].

Our dataset indicated a higher diversity of RVB genotypes in swine hosts compared to other hosts, which has been observed in swine RVC as well. Percent identities of swine RVC VP7 are notably lower than human and bovine strains, and greater numbers of genotypes of nearly all gene segments exist for swine RVC than in other hosts [40,43,44]. This highlights the important contribution of swine to the genetic diversity of RVB and RVC and, as others have suggested, may indicate swine are the main hosts for these viruses [44]. While the range of VP6 percent identities found in this study agrees with previous work, we found that only sequences of porcine origin had percent identities lower than 70%, and it is possible that rotaviruses evolve more heterogeneously in swine than in other hosts.

Reassortment among rotaviruses is a common phenomenon due to their segmented genomes [45,46]. A previous study investigated the VP6 and VP7 segments among many of the reference porcine strains used in this study and found frequent reassortment [31]. Even within genotypes, substantial genetic diversity can be present, and reassortment among sub-clades within human-specific RVB genotypes [47]. In the samples sequenced for this study, we found evidence of frequent VP4 segment reassortment, which is likely due to coinfection of RVB within swine. Reassortment of the outer capsid VP7 and VP4 proteins, in particular, would be expected to confer an evolutionary advantage since they are the targets of neutralization and reassortment help strains escape immune recognition.

Phylogenetic analysis exhibited host-specific RVB genotypes for murine, human and porcine species, and genotype constellations for these species did not show cross-species reassortment events, which is in contrast to RVA where multiple interspecies events were reported, especially between humans and domestic animals such as swine, bovine, and horses [29,48,49]. Human–porcine and bovine–porcine reassortment of the VP3 and VP6 genes was reported in RVC [40]. The exception to the RVB host specificity found in this study was the phylogenetic clustering of bovine and caprine RVB strains [24]. Whole genome sequencing of goat RVA strains reveal close phylogenetic relationship with bovine strains, pointing to historical reassortment events between the two host species [50,51,52]. Interspecies transmission of RVB could have occurred to produce this genetic similarity, although the bovine and goat strains were geographically separated, and any interspecies reassortment probably happened many years ago. Although we did not observe geographical separation of genotypes, additional sequencing and epidemiological studies could elucidate prevalence of genotypes in other countries.

In summary, a provisional genome-based classification for RVB strains from human, bovine, caprine, porcine and murine species was established, providing relevant information to understanding the evolution and epidemiology of RVB. Future research should include the sequencing and analysis of more RVB strains to ensure the consistency of the nucleotide cutoff values, remaining the true diversity of RVB.

## Figures and Tables

**Table 1:** Ranges of nucleotide percent identities by host species and gene segment.

Gene Segment	Porcine		Bovine		Human	
	Range	Median	Range	Median	Range	Median
VP7	55.0–100	74.0 ^b^	77.0–100	94.0 ^a^	91.0–100	98.0 ^c^
VP4	64.0–100	75.5 ^a^	81.0–100	82.0 ^ab^	90.0–100	98.0 ^b^
VP6	63.0–100	77.0 ^b^	82.0–100	87.0 ^a^	92.0–100	98.0 ^a^
VP1	78.0–100	100.0 ^a^	94.0–100	94.0 ^ab^	90.0–100	98.0 ^b^
VP2	82.0–100	100 ^a^	77.0–100	77.0 ^ab^	90.0–100	98.0 ^b^
VP3	72.0–100	97.0 ^b^	92.0–100	99.0 ^a^	90.0–100	98.0 ^a^
NSP1	67.0–100	83.0 ^a^	71.0–100	98 ^a^	91.0–100	98.0 ^b^
NSP2	66.0–100	86.0 ^a^	82.0–100	89.0 ^ab^	93.0–100	99.0 ^b^
NSP3	58.0–100	100 ^b^	94.0–100	99 ^a^	89.0–100	98.0 ^a^
NSP4	77.0–100	93.0 ^b^	96.0–100	99.0 ^a^	90.0–100	98.0 ^a^
NSP5	44.0–100	81.0 ^b^	77.0–100	94.0 ^a^	89.0–100	97.0 ^c^

Values with superscripts “a”, “b”, and “c” are statistically different from one another (*p*-value < 0.05) within the same gene segment based on a Kruskal–Wallis rank sum test.

**Table 2:** Proposed nucleotide cutoff values and genotypes for rotavirus B whole genome classification.

Gene Segment	Number of Sequences	Previously Proposed Nucleotide Cutoff	Reference	Currently Proposed Nucleotide Cutoff	Genotypes in Each Host Species				
					Murine	Human	Bovine	Caprine	Porcine
VP7	419	80%	Marthaler et al., 2012	80%	G1	G2	G3, G5	G3	G4, G6–G26
VP4	64	--	--	80%	P[1]	P[2]	P[3]	P[3]	P[4], P[5]
VP6	144	81%	Marthaler et al., 2014	81%	I1	I2	I3	I3	I4–I13
VP1	54	--	--	78%	R1	R2	R5	R3	R4
VP2	57	--	--	79%	C1	C2	C3, C5	C3	C4
VP3	61	70%	Hayashi-Miyamoto et al., 2017	77% *	M1	M2	M3	M3	M4, M5
NSP1	68	76%	Suzuki et al., 2011	76%	A1	A2	A4, A5	A3, A4	A6–A8
NSP2	89	75%	Suzuki et al., 2012	83% *	N1	N2	N3, N4	N3	N5–N10
NSP3	58	78%	Hayashi-Miyamoto et al., 2017	78%	T1	T2	T3	T3	T4–T6
NSP4	68	70%	Hayashi-Miyamoto et al., 2017	76% *	E1	E2	E3	E3	E4
NSP5	95	78%	Suzuki et al., 2012	79% *	H1	H2	H3, H5	H3	H4, H6, H7

* Indicates cutoff value different from previously proposed value.

**Table 3:** Genome constellations for RVB strains with at least 6 gene segments.

	VP7	VP4	VP6	VP1	VP2	VP3	NSP1	NSP2	NSP3	NSP4	NSP5
RVB/Rat-wt/USA/IDIR/XXXX	G1	P[1]	I1	R1	C1	M1	A1	N1	T1	E1	H1
RVB/Human-wt ^	G2	P[2]	I2	R2	C2	M2	A2	N2	T2	E2	H2
RVB/Bovine-wt/IND/DB176/2001	G5	P[3]	I3	R5	C5	na	A5	N4	na	na	H5
RVB/Bovine-wt/IND/RUBV226/2004	G5	P[3]	I3	R5	C5	na	A5	N4	na	na	na
RVB/Bovine-wt/IND/RUBV282/2005	G5	P[3]	I3	R5	C5	na	A5	N4	na	na	H5
RVB/Bovine-wt/JPN/G-2006/2006/G3PX	G3	P[3]	na	na	na	M3	A3	N3	T3	E3	H3
RVB/Bovine-wt/JPN/IS-1/1999/G3PX	G3	P[3]	*	*	na	M3	A3	*	T3	E3	na
RVB/Bovine-wt/JPN/IS-2/2002/G3PX	G3	P[3]	*	*	C3	M3	na	na	T3	E3	na
RVB/Goat-wt/USA/CA22/2014	G3	P[3]	I3	na	C3	na	A3	N3	T3	E3	H3
RVB/Goat-wt/USA/Minnesota-1/2016	G3	P[3]	I3	R3	C3	M3	A3	N3	T3	E3	H3
RVB/Pig-tc/USA/LS00011_Ohio/XXXX/GXP[X]	G12	P[4]	I13	R4	C4	M4	A8	N10	T6	E4	H7
RVB/Pig-wt/USA/IL10/2013 & IL10B/2013	G16	P[4]/P[5]	I13	R4	C4	M4	A8	N10	T4	E4	H7
RVB/Pig-wt/USA/IL11/2013	G16	P[4]	I13	R4	C4	M4	A8	N10	T4	E4	H7
RVB/Pig-wt/USA/IL12/2013 & IL12B/2013	G16	P[4]/P[5]	I13	R4	C4	M4	A8	N10	T4	E4	H7
RVB/Pig-wt/USA/IL13/2013	G16	P[4]	I13	R4	C4	M4	A8	N10	T4	E4	H7
RVB/Pig-wt/USA/IL14/2013 & IL14B/2013	G16	P[4]/P[5]	I13	R4	C4	M4	A8	N10	T4	E4	H7
RVB/Pig-wt/USA/IL15/2013 & L15B/2013	G16	P[4]/P[5]	I13	R4	C4	M4	A8	N10	T4	E4	H7
RVB/Pig-wt/USA/IL16/2013	G16	P[5]	I13	R4	C4	M4	A8	N10	T4	E4	H7
RVB/Pig-wt/USA/IL17/2013	G16	P[5]	I13	R4	C4	M4	A8	N10	T4	E4	H7
RVB/Pig-wt/USA/IL18/2013	G16	P[5]	I13	R4	C4	M4	A8	N10	T4	E4	H7
RVB/Pig-wt/USA/IL19/2013	G16	P[5]	I13	R4	C4	M4	A8	N10	T4	E4	H7
RVB/Pig-wt/USA/IL20/2013	G16	P[5]	I13	R4	C4	M4	A8	N10	T4	E4	H7
RVB/Pig-wt/USA/IL21/2013	G16	P[5]	I13	R4	C4	M4	A8	N10	T4	E4	H7
RVB/Pig-wt/USA/IL3/2012	G16	P[5]	I13	R4	C4	M4	A8	N10	T4	E4	H7
RVB/Pig-wt/USA/IL4/2012	G16	P[5]	I13	R4	C4	M4	A8	N10	T4	E4	H7
RVB/Pig-wt/USA/IL5/2012	G16	P[4]	I13	R4	C4	M4	A8	N10	T4	E4	H7
RVB/Pig-wt/USA/IL6/2012	G16	P[5]	I13	R4	C4	M4	A8	N10	T4	E4	H7
RVB/Pig-wt/USA/IL7/2012	G16	P[4]	I13	R4	C4	M4	A8	N10	T4	E4	H7
RVB/Pig-wt/USA/IL8/2013 & IL8B/2013	G16	P[4]/P[5]	I13	R4	C4	M4	A8	N10	T4	E4	H7
RVB/Pig-wt/USA/IL9/2013	G16	P[5]	I13	R4	C4	M4	A8	N10	T4	E4	H7
RVB/Pig-wt/USA/KS1/2012	G16	P[4]	I13	R4	C4	M4	A8	N10	T5	E4	H7
RVB/Pig-wt/USA/KS2/2012 & KS2B/2012	G14/G16	na	I13	R4	na	M4	A8	N10	T5	E4	H7

* indicates partial sequences with less than 80% of the ORF that were not included in the analysis. ^ indicates all human strains. Orange indicates pig RVB genes, Green indicates bovine and goat RVB gene segments. na: not available.

## Supplementary Materials

The following are available online at http://www.mdpi.com/2076-0817/7/2/44/s1, Supplemental Figure S1: Nucleotide pairwise identity frequency graphs of rotavirus B genes.

## References

[1] Saif L.J., Jiang B. (1994). Nongroup A rotaviruses of humans and animals. Curr. Top. Microbiol. Immunol..

[2] Martella V., Bányai K., Matthijnssens J., Buonavoglia C., Ciarlet M. (2010). Zoonotic aspects of rotaviruses. Vet. Microbiol..

[3] Estes M.K., Greenberg H.B. (2013). Rotaviruses, Fields Virology.

[4] Matthijnssens J., Otto P.H., Ciarlet M., Desselberger U., Van Ranst M., Johne R. (2012). VP6-sequence-based cutoff values as a criterion for rotavirus species demarcation. Arch. Virol..

[5] Mihalov-Kovács E., Gellért Á., Marton S., Farkas S.L., Fehér E., Oldal M., Jakab F., Martella V., Bányai K. (2015). Candidate new rotavirus species in sheltered dogs, Hungary. Emerg. Infect. Dis..

[6] Bányai K., Kemenesi G., Budinski I., Földes F., Zana B., Marton S., Varga-Kugler R., Oldal M., Kurucz K., Jakab F. (2017). Candidate new rotavirus species in Schreiber’s bats, Serbia. Infect. Genet. Evol. J. Mol. Epidemiol. Evol. Genet. Infect. Dis..

[7] Matthijnssens J., Martella V., Van Ranst M. (2010). Genomic evolution, host-species barrier, reassortment and classification of rotaviruses. Future Virol..

[8] Hung T., Chen G.M., Wang C.G., Yao H.L., Fang Z.Y., Chao T.X., Chou Z.Y., Ye W., Chang X.J., Den S.S. (1984). Waterborne outbreak of rotavirus diarrhoea in adults in China caused by a novel rotavirus. Lancet Lond. Engl..

[9] Chen C.M., Hung T., Bridger J.C., McCrae M.A. (1985). Chinese adult rotavirus is a group B rotavirus. Lancet Lond. Engl..

[10] Krishnan T., Sen A., Choudhury J.S., Das S., Naik T.N., Bhattacharya S.K. (1999). Emergence of adult diarrhoea rotavirus in Calcutta, India. Lancet Lond. Engl..

[11] Kelkar S.D., Zade J.K. (2004). Group B rotaviruses similar to strain CAL-1, have been circulating in Western India since 1993. Epidemiol. Infect..

[12] Sanekata T., Ahmed M.U., Kader A., Taniguchi K., Kobayashi N. (2003). Human group B rotavirus infections cause severe diarrhea in children and adults in Bangladesh. J. Clin. Microbiol..

[13] Aung T.S., Kobayashi N., Nagashima S., Ghosh S., Aung M.S., Oo K.Y., Win N. (2009). Detection of group B rotavirus in an adult with acute gastroenteritis in Yangon, Myanmar. J. Med. Virol..

[14] Joshi M.S., Ganorkar N.N., Ranshing S.S., Basu A., Chavan N.A., Gopalkrishna V. (2017). Identification of group B rotavirus as an etiological agent in the gastroenteritis outbreak in Maharashtra, India. J. Med. Virol..

[15] Alam M.M., Pun S.B., Gauchan P., Yokoo M., Doan Y.H., Tran T.N.H., Nakagomi T., Nakagomi O., Pandey B.D. (2013). The First Identification of Rotavirus B from Children and Adults with Acute Diarrhoea in Kathmandu, Nepal. Trop. Med. Health.

[16] Eiden J.J., Nataro J., Vonderfecht S., Petric M. (1992). Molecular cloning, sequence analysis, in vitro expression, and immunoprecipitation of the major inner capsid protein of the IDIR strain of group B rotavirus (GBR). Virology.

[17] Barman P., Ghosh S., Das S., Varghese V., Chaudhuri S., Sarkar S., Krishnan T., Bhattacharya S.K., Chakrabarti A., Kobayashi N. (2004). Sequencing and sequence analysis of VP7 and NSP5 genes reveal emergence of a new genotype of bovine group B rotaviruses in India. J. Clin. Microbiol..

[18] Chang K.O., Parwani A.V., Smith D., Saif L.J. (1997). Detection of group B rotaviruses in fecal samples from diarrheic calves and adult cows and characterization of their VP7 genes. J. Clin. Microbiol..

[19] Chinsangaram J., Schore C.E., Guterbock W., Weaver L.D., Osburn B.I. (1995). Prevalence of group A and group B rotaviruses in the feces of neonatal dairy calves from California. Comp. Immunol. Microbiol. Infect. Dis..

[20] Ghosh S., Varghese V., Sinha M., Kobayashi N., Naik T.N. (2007). Evidence for interstate transmission and increase in prevalence of bovine group B rotavirus strains with a novel VP7 genotype among diarrhoeic calves in Eastern and Northern states of India. Epidemiol. Infect..

[21] Hayashi M., Murakami T., Kuroda Y., Takai H., Ide H., Awang A., Suzuki T., Miyazaki A., Nagai M., Tsunemitsu H. (2016). Reinfection of adult cattle with rotavirus B during repeated outbreaks of epidemic diarrhea. Can. J. Vet. Res..

[22] Tsunemitsu H., Morita D., Takaku H., Nishimori T., Imai K., Saif L.J. (1999). First detection of bovine group B rotavirus in Japan and sequence of its VP7 gene. Arch. Virol..

[23] Shen S., McKee T.A., Wang Z.D., Desselberger U., Liu D.X. (1999). Sequence analysis and in vitro expression of genes 6 and 11 of an ovine group B rotavirus isolate, KB63: Evidence for a non-defective, C-terminally truncated NSP1 and a phosphorylated NSP5. J. Gen. Virol..

[24] Chen F., Knutson T.P., Ciarlet M., Sturos M., Marthaler D.G. (2018). Complete genome characterization of a rotavirus B (RVB) strain identified in Alpine goat kids with enteritis reveals inter-species transmission with RVB bovine strains. J. Gen. Virol..

[25] Theil K.W., Grooms D.L., McCloskey C.M., Redman D.R. (1995). Group B rotavirus associated with an outbreak of neonatal lamb diarrhea. J. Vet. Diagn. Investig. Off. Publ. Am. Assoc. Vet. Lab. Diagn. Inc.

[26] Chasey D., Bridger J.C., McCrae M.A. (1986). A new type of atypical rotavirus in pigs. Arch. Virol..

[27] Kuga K., Miyazaki A., Suzuki T., Takagi M., Hattori N., Katsuda K., Mase M., Sugiyama M., Tsunemitsu H. (2009). Genetic diversity and classification of the outer capsid glycoprotein VP7 of porcine group B rotaviruses. Arch. Virol..

[28] Marthaler D., Rossow K., Gramer M., Collins J., Goyal S., Tsunemitsu H., Kuga K., Suzuki T., Ciarlet M., Matthijnssens J. (2012). Detection of substantial porcine group B rotavirus genetic diversity in the United States, resulting in a modified classification proposal for G genotypes. Virology.

[29] Matthijnssens J., Ciarlet M., Heiman E., Arijs I., Delbeke T., McDonald S.M., Palombo E.A., Iturriza-Gómara M., Maes P., Patton J.T. (2008). Full genome-based classification of rotaviruses reveals a common origin between human Wa-Like and porcine rotavirus strains and human DS-1-like and bovine rotavirus strains. J. Virol..

[30] Hayashi-Miyamoto M., Murakami T., Minami-Fukuda F., Tsuchiaka S., Kishimoto M., Sano K., Naoi Y., Asano K., Ichimaru T., Haga K. (2017). Diversity in VP3, NSP3, and NSP4 of rotavirus B detected from Japanese cattle. Infect. Genet. Evol. J. Mol. Epidemiol. Evol. Genet. Infect. Dis..

[31] Marthaler D., Suzuki T., Rossow K., Culhane M., Collins J., Goyal S., Tsunemitsu H., Ciarlet M., Matthijnssens J. (2014). VP6 genetic diversity, reassortment, intragenic recombination and classification of rotavirus B in American and Japanese pigs. Vet. Microbiol..

[32] Suzuki T., Soma J., Kuga K., Miyazaki A., Tsunemitsu H. (2012). Sequence and phylogenetic analyses of nonstructural protein 2 genes of species B porcine rotaviruses detected in Japan during 2001–2009. Virus Res..

[33] Suzuki T., Soma J., Miyazaki A., Tsunemitsu H. (2012). Phylogenetic analysis of nonstructural protein 5 (NSP5) gene sequences in porcine rotavirus B strains. Infect. Genet. Evol. J. Mol. Epidemiol. Evol. Genet. Infect. Dis..

[34] Suzuki T., Kuga K., Miyazaki A., Tsunemitsu H. (2011). Genetic divergence and classification of non-structural protein 1 among porcine rotaviruses of species B. J. Gen. Virol..

[35] Lahon A., Ingle V.C., Birade H.S., Raut C.G., Chitambar S.D. (2014). Molecular characterization of group B rotavirus circulating in pigs from India: Identification of a strain bearing a novel VP7 genotype, G21. Vet. Microbiol..

[36] Lambden P.R., Cooke S.J., Caul E.O., Clarke I.N. (1992). Cloning of noncultivatable human rotavirus by single primer amplification. J. Virol..

[37] Maan S., Rao S., Maan N.S., Anthony S.J., Attoui H., Samuel A.R., Mertens P.P.C. (2007). Rapid cDNA synthesis and sequencing techniques for the genetic study of bluetongue and other dsRNA viruses. J. Virol. Methods.

[38] Kearse M., Moir R., Wilson A., Stones-Havas S., Cheung M., Sturrock S., Buxton S., Cooper A., Markowitz S., Duran C. (2012). Geneious Basic: An integrated and extendable desktop software platform for the organization and analysis of sequence data. Bioinformatics.

[39] Guindon S., Dufayard J.-F., Lefort V., Anisimova M., Hordijk W., Gascuel O. (2010). New algorithms and methods to estimate maximum-likelihood phylogenies: Assessing the performance of PhyML 3.0. Syst. Biol..

[40] Suzuki T., Hasebe A. (2017). A provisional complete genome-based genotyping system for rotavirus species C from terrestrial mammals. J. Gen. Virol..

[41] Ghosh S., Navarro R., Malik Y.S., Willingham A.L., Kobayashi N. (2015). Whole genomic analysis of a porcine G6P[13] rotavirus strain. Vet. Microbiol..

[42] Vlasova A.N., Amimo J.O., Saif L.J. (2017). Porcine Rotaviruses: Epidemiology, Immune Responses and Control Strategies. Viruses.

[43] Jeong Y.-J., Matthijnssens J., Kim D.-S., Kim J.-Y., Alfajaro M.M., Park J.-G., Hosmillo M., Son K.-Y., Soliman M., Baek Y.-B. (2015). Genetic diversity of the VP7, VP4 and VP6 genes of Korean porcine group C rotaviruses. Vet. Microbiol..

[44] Marthaler D., Rossow K., Culhane M., Collins J., Goyal S., Ciarlet M., Matthijnssens J. (2013). Identification, phylogenetic analysis and classification of porcine group C rotavirus VP7 sequences from the United States and Canada. Virology.

[45] McDonald S.M., Matthijnssens J., McAllen J.K., Hine E., Overton L., Wang S., Lemey P., Zeller M., Van Ranst M., Spiro D.J. (2009). Evolutionary Dynamics of Human Rotaviruses: Balancing Reassortment with Preferred Genome Constellations. PLoS Pathog..

[46] Steyer A., Poljsak-Prijatelj M., Barlic-Maganja D., Marin J. (2008). Human, porcine and bovine rotaviruses in Slovenia: Evidence of interspecies transmission and genome reassortment. J. Gen. Virol..

[47] Aung M.S., Nahar S., Aida S., Paul S.K., Hossain M.A., Ahmed S., Haque N., Ghosh S., Malik Y.S., Urushibara N. (2017). Distribution of two distinct rotavirus B (RVB) strains in the north-central Bangladesh and evidence for reassortment event among human RVB revealed by whole genomic analysis. Infect. Genet. Evol..

[48] Agbemabiese C.A., Nakagomi T., Gauchan P., Sherchand J.B., Pandey B.D., Cunliffe N.A., Nakagomi O. (2017). Whole genome characterisation of a porcine-like human reassortant G26P[19] Rotavirus A strain detected in a child hospitalised for diarrhoea in Nepal, 2007. Infect. Genet. Evol..

[49] Pietsch C., Liebert U.G. (2018). Molecular characterization of different equine-like G3 rotavirus strains from Germany. Infect. Genet. Evol..

[50] Bwogi J., Jere K.C., Karamagi C., Byarugaba D.K., Namuwulya P., Baliraine F.N., Desselberger U., Iturriza-Gomara M. (2017). Whole genome analysis of selected human and animal rotaviruses identified in Uganda from 2012 to 2014 reveals complex genome reassortment events between human, bovine, caprine and porcine strains. PLoS ONE.

[51] Ghosh S., Alam M.M., Ahmed M.U., Talukdar R.I., Paul S.K., Kobayashi N. (2010). Complete genome constellation of a caprine group A rotavirus strain reveals common evolution with ruminant and human rotavirus strains. J. Gen. Virol..

[52] Louge Uriarte E.L., Badaracco A., Matthijnssens J., Zeller M., Heylen E., Manazza J., Miño S., Van Ranst M., Odeón A., Parreño V. (2014). The first caprine rotavirus detected in Argentina displays genomic features resembling virus strains infecting members of the Bovidae and Camelidae. Vet. Microbiol..

