# Supplementary material for: Whole Genome Classification and Phylogenetic Analyses of Rotavirus B strains from the United States
Source: Pathogens. 2018 Apr 18;7(2):44. doi: 10.3390/pathogens7020044 (PMC6027208; doi:10.3390/pathogens7020044)
Supplement: Supplementary file 1 [file pathogens-07-00044-s001.pdf]

Figure S1. Nucleotide pairwise identity frequency graphs of rotavirus B genes. The cutoff values are represented by dashed blue lines.

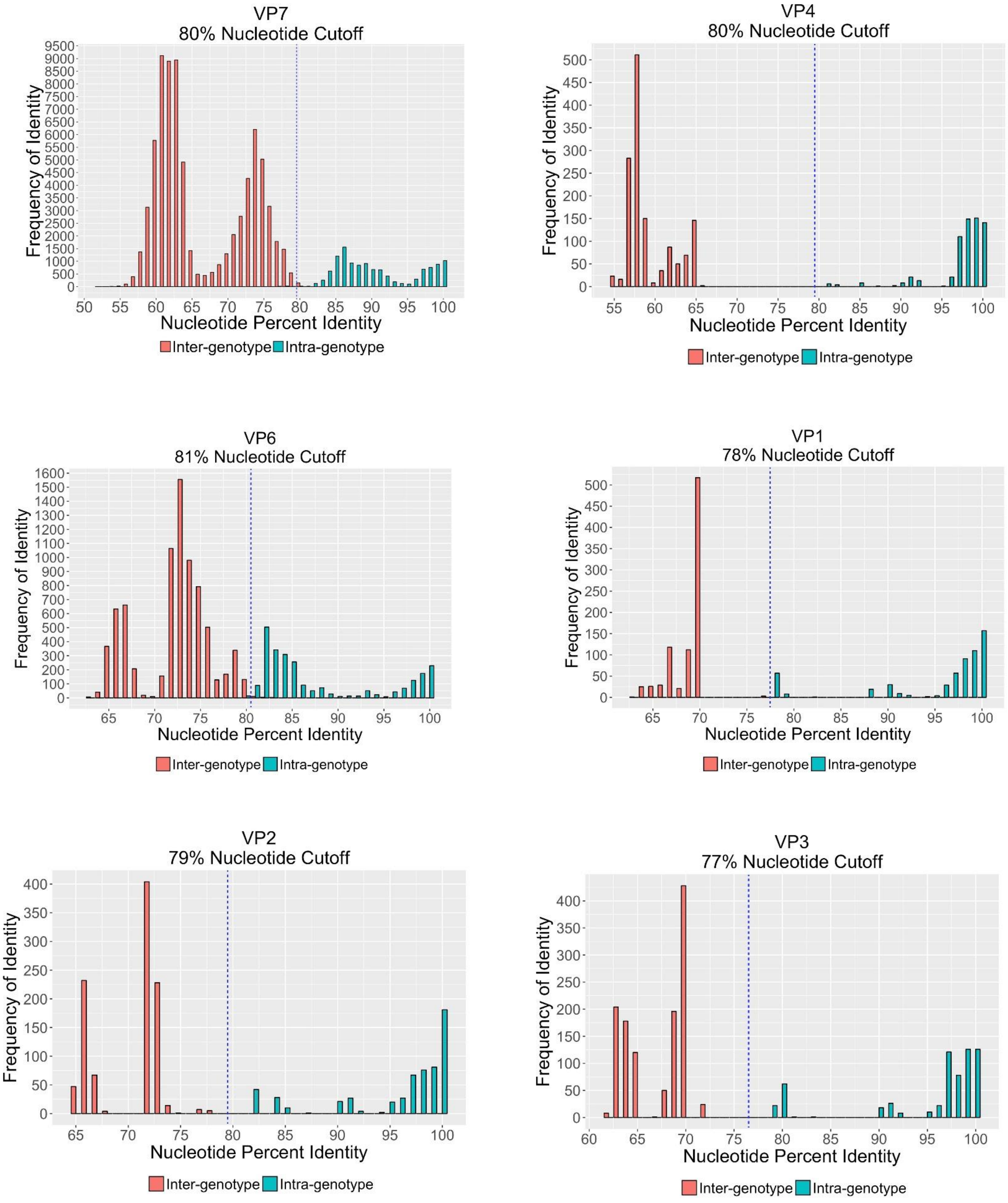

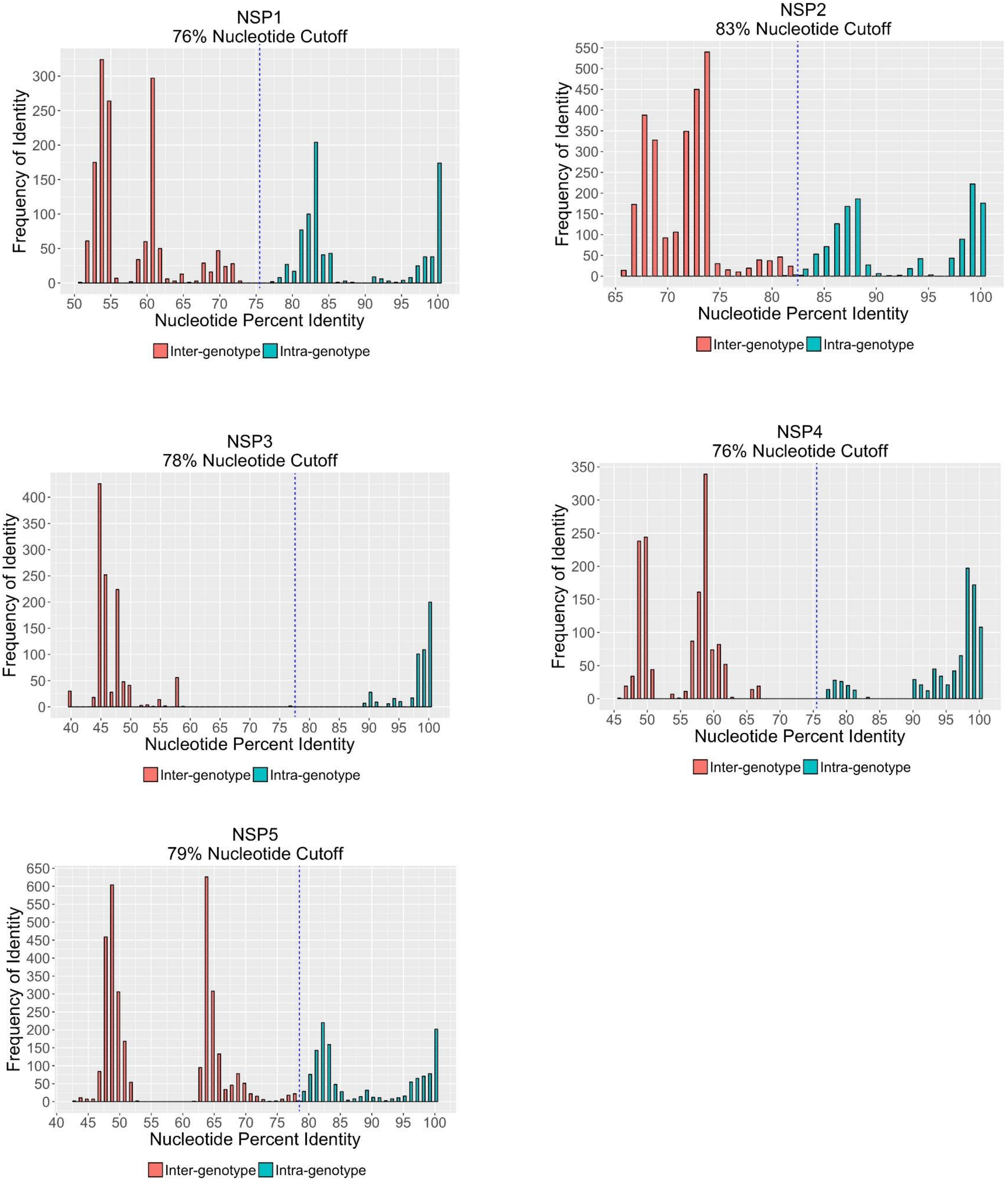
NSP1
76% Nucleotide Cutoff
Frequency of Identity
Nucleotide Percent Identity
Inter-genotype
Intra-genotype
NSP2
83% Nucleotide Cutoff
Frequency of Identity
Nucleotide Percent Identity
Inter-genotype
Intra-genotype
NSP3
78% Nucleotide Cutoff
Frequency of Identity
Nucleotide Percent Identity
Inter-genotype
Intra-genotype
NSP4
76% Nucleotide Cutoff
Frequency of Identity
Nucleotide Percent Identity
Inter-genotype
Intra-genotype
NSP5
79% Nucleotide Cutoff
Frequency of Identity
Nucleotide Percent Identity
Inter-genotype
Intra-genotype
